# Trialstreamer: A living, automatically updated database of clinical trial reports

**DOI:** 10.1093/jamia/ocaa163

**Published:** 2020-09-17

**Authors:** Iain J Marshall, Benjamin Nye, Joël Kuiper, Anna Noel-Storr, Rachel Marshall, Rory Maclean, Frank Soboczenski, Ani Nenkova, James Thomas, Byron C Wallace

**Affiliations:** o1 School of Population Health and Environmental Sciences, King’s College London, London, United Kingdom; o2 Khoury College of Computer Sciences, Northeastern University, Boston, Massachusetts, USA; o3 Vortext Systems, Groningen, the Netherlands; o4 Cochrane Dementia Group, University of Oxford, Oxford, United Kingdom; o5 Cochrane Editorial and Methods Department, London, United Kingdom; o6 Computer and Information Science, University of Pennsylvania, Philadelphia, Pennsylvania, USA; o7 EPPI-Centre, UCL Social Research Institute, University College London, London, United Kingdom

**Keywords:** randomized controlled trials, research synthesis, automatic database curation, evidence based medicine

## Abstract

**Objective:**

Randomized controlled trials (RCTs) are the gold standard method for evaluating whether a treatment works in health care but can be difficult to find and make use of. We describe the development and evaluation of a system to automatically find and categorize all new RCT reports.

**Materials and Methods:**

Trialstreamer continuously monitors PubMed and the World Health Organization International Clinical Trials Registry Platform, looking for new RCTs in humans using a validated classifier. We combine machine learning and rule-based methods to extract information from the RCT abstracts, including free-text descriptions of trial PICO (populations, interventions/comparators, and outcomes) elements and map these snippets to normalized MeSH (Medical Subject Headings) vocabulary terms. We additionally identify sample sizes, predict the risk of bias, and extract text conveying key findings. We store all extracted data in a database, which we make freely available for download, and via a search portal, which allows users to enter structured clinical queries. Results are ranked automatically to prioritize larger and higher-quality studies.

**Results:**

As of early June 2020, we have indexed 673 191 publications of RCTs, of which 22 363 were published in the first 5 months of 2020 (142 per day). We additionally include 304 111 trial registrations from the International Clinical Trials Registry Platform. The median trial sample size was 66.

**Conclusions:**

We present an automated system for finding and categorizing RCTs. This yields a novel resource: a database of structured information automatically extracted for all published RCTs in humans. We make daily updates of this database available on our website (https://trialstreamer.robotreviewer.net).

## INTRODUCTION

### Background and significance

Randomized controlled trials (RCTs) represent the gold standard method for determining what interventions work in health care,^1^ and access to the results of such trials is central to the practice of evidence-based medicine. However, finding and making use of RCT evidence can be difficult.^2^ The number of RCTs published accelerates every year,^3^ but they still make up a tiny fraction of the contents of health research databases.^4^ It is therefore difficult to retrieve trials relevant to particular clinical questions.

An up-to-date resource comprising all published RCTs and data on ongoing trials would facilitate efficient retrieval of the best available evidence, especially if it allowed for structured search with respect to individual PICO (study populations, interventions/comparators, and outcomes) elements.^5^ This would be a boon to healthcare practitioners and to researchers interested in seeking the latest evidence for a given question at the point of care, or in gaining a broad overview of all clinical evidence on particular topics (eg, as in a scoping review or similar evidence mapping exercise). In this article, we describe our development of such a system, which we call Trialstreamer. Using a validated machine learning model,^4^ Trialstreamer identifies articles and registrations of new RCTs in humans as they are published, and runs these through a suite of trained data extraction models to extract elements of interest, including study sample sizes, key findings, descriptions of the PICO elements, and an indicator of the risk of bias.

There is a burgeoning literature on the use of machine learning to automatically classify and interpret biomedical research articles, and a number of discrete tasks have been accurately automated.^6^ These include automated classification of articles by study method^4^^,^^7^^,^^8^ and whether they are conducted in humans,^9^ among others. Data extraction systems have been created that can automatically retrieve unstructured information on the trial PICO elements,^10–12^ and risk of bias.^13–16^ Here, we build on these works, creating and validating a pipeline of text classification and extraction models to produce a real-time evidence surveillance system.

We store all identified RCT records with their associated extracted data in a publicly available, continuously updated database. We believe that this will be a valuable resource to the larger informatics community, in addition to healthcare professionals and health researchers. We also make the data accessible via an open-source prototype web interface (https://trialstreamer.robotreviewer.net/). This interface capitalizes on the extracted data to allow users to precisely structure queries to address a clinical question of interest, and to automatically rank retrieved articles to prioritize the largest and highest-quality trials.

## OBJECTIVE

We describe the Trialstreamer database, the elements it comprises, and how we keep the database up to date. We review the machine learning models that we use to extract individual elements from trial reports, and we empirically evaluate the accuracy of the system components. We make the data freely accessible in several ways: via our prototype web portal, by bulk data download from the open science platform Zenodo (https://doi.org/10.5281/zenodo.3767068), or by executing the Trialstreamer source code locally to reproduce the database.

## MATERIALS AND METHODS

### Overview

**Figure 1.**
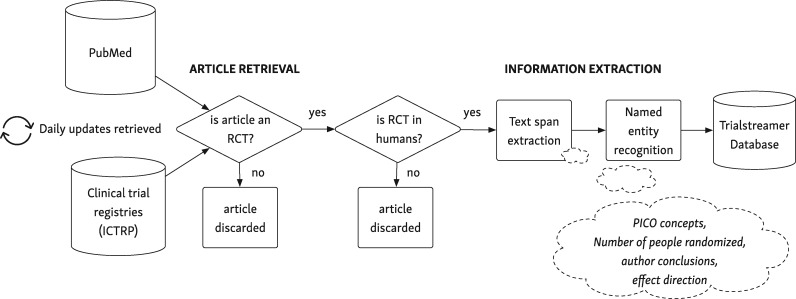
How articles are retrieved, annotated, and stored. ICTRP: International Clinical Trials Registry Platform; PICO: populations, interventions/comparators, and outcomes; RCT: randomized controlled trial.

Trialstreamer monitors key research databases, seeking to retrieve reports of RCTs in humans quickly after publication. The architecture of the system is summarized in Figure 1 and Table 1. First, we collect peer-reviewed journal articles (via PubMed), and registrations of ongoing trials (via the World Health Organization International Clinical Trials Registry Platform [ICTRP]) (during the current COVID-19 [coronavirus disease 2019] pandemic, we additionally monitor preprints of COVID-19 trials from *medRxiv*). ICTRP aggregates data from 17 international clinical trial registers, including ClinicalTrials.gov. From these sources, we retrieve all research articles and study registrations indexed in PubMed or ICTRP. We then use a machine learning system to classify the study design of each record, as to whether it describes an RCT or not. Records classified as RCTs in humans are retained, and other study designs are discarded. This step should discard >95% of the source articles. Next, key data are extracted from the RCT abstracts (namely a description of the PICO, the number of participants, a snippet stating the main findings, and an assessment of the risk of bias). The articles, together with the extracted information items, are saved to a relational database. The full process is quick: articles are typically available in the database (with full annotations) less than an hour after publication at the source. The updated database is accessible via a web search portal, and also for bulk download. We provide an overview of the machine learning systems for each step subsequently. All code for generating the database and the machine learning models is freely available via our website (https://trialstreamer.robotreviewer.net). We describe each step in detail subsequently.

**Table 1.** Summary of Trialstreamer components

Component	Model architecture	How used in Trialstreamer	Data
RCT vs non-RCT study classifier	Ensemble of support vector machine and convolutional neural network models^4^	Previously validated model^4^ used with new “balanced” classification threshold	Training and parameter tuning on 280 000 abstracts with crowdsourced labels from Cochrane Crowd Evaluation on 49,028 abstracts manually labeled by the Clinical Hedges team
Human vs non-human study classifier	Support vector machine model, based on Cohen et al^9^	New model trained and validated, based on prior method	467 153 abstracts of RCTs from PubMed, using MeSH term “Humans” Training and parameter tuning on 75% of data, evaluation on withheld 25% of data
Sample size extraction	Multilayer perceptron model for classifying integers in abstracts	New model trained and validated	Trained on 8935 abstracts with sample sizes (6315 taken from structured results data in ClinicalTrials.gov; 2620 manually labelled by our team) Evaluation on 500 separate abstracts manually labelled by the authors
PICO text spans	LSTM-CRF model^12^	Previously validated model used unchanged^12^	5000 abstracts with from *EBM-NLP* dataset^12^ Model training/parameter tuning on 4,800 abstracts labeled by lay crowd workers. Evaluation on 200 withheld abstracts labeled by medical doctors
PICO concepts	Rule-based concept extraction, following the method of *Metamap Lite* by Demner-Fushman et al^17^	Reimplementation of previously described method	972 371 selected concepts (CUIs) and their associated text from the UMLS Metathesaurus 2019 edition. Concepts included were from the source vocabularies: SNOMED CT, RxNorm, MeSH, MedDRA, and the World Health Organization ATC classification system Evaluation on 1497 unseen abstracts with crowd-sourced PICO MeSH labels from Cochrane Crowd
Risk of bias	Logistic regression model with L2 regularization, using bag-of-words representation (unigrams, bigrams and trigrams) of the title and abstract text to generate an overall score	New model trained and validated	13 463 abstracts of RCTs with Cochrane Risk of Bias tool assessments in the Cochrane Library: 60% used for training; 40% withheld for evaluation

ATC: Anatomic Therapeutic Chemical; CUI: concept unique identifier; LSTM-CRF: long short-term memory conditional random fields; MedDRA: Medical Dictionary for Regulatory Activities; MeSH: Medical Subject Headings; RCT: randomized controlled trial; SNOMED CT: Systematized Nomenclature of Medicine Clinical Terms; UMLS: Unified Medical Language System.

### Article retrieval

#### Identifying RCTs

PubMed indexes >30 million research articles (https://pubmed.ncbi.nlm.nih.gov/), and the number of articles is growing rapidly. We are interested in the subset of these articles that describe RCTs, and more specifically, RCTs conducted in humans. Such articles comprise 1%-2% of the total.^4^ While we could use the high-quality manual indexes applied by PubMed staff (namely, the “Randomized Controlled Trial” Publication Type, and the “Humans” Medical Subject Headings [MeSH] term), there is several months’ lag from publication date to indexing, given that this is a manual process. This would result in our system failing to include recently published material.

Instead, we use a machine learning classifier that we have described previously to continuously identify new RCT reports (MeSH refers to the Medical Subject Headings vocabulary, maintained by the National Library of Medicine and used for indexing articles in the MEDLINE database).^4^ In short, this system uses an ensemble of models comprising a support vector machine and 10 independent convolutional neural network models. These models were trained on a set of 280 000 abstracts manually labeled by the Cochrane Crowd project (https://crowd.cochrane.org/). For articles that have a manually applied publication type, we incorporate this information as an additional input feature for the ensemble.

Our prior work focused on classifying RCTs for systematic review searches; such reviews emphasize finding all relevant studies. Therefore, for that application, we designed the classifier to have very high recall (near 99%), which as a trade-off corresponded to relatively low precision (≈20%). The assumption here is that the model serves as an initial filter, and that the output would then go through a manual screening step, such that the remaining irrelevant articles would be excluded.

By contrast, Trialstreamer seeks to be useful for clinical question answering and literature scoping; it is intended to be used fully automatically, without a manual screening step. We therefore calibrated and evaluated a new classification threshold that seeks a more appropriate balance of recall and precision for this use. To achieve this, we drew receiver-operating characteristic curves for the trained models with regard to a set of 49 028 abstracts labeled by the McMaster University Clinical Hedges team.^18^ We set a threshold which maximized the sum of sensitivity and specificity (ie, the outer left extreme of the curve). To evaluate the performance of this strategy, we conducted 5000 bootstrap iterations, in which a classification threshold was set on a random sample with replacement of the Hedges dataset, and evaluated on the data not included in that sample.^19^ We evaluated the accuracy of binary prediction (precision/recall) and calibration (via calibration curves, Brier scores, and the C-statistic). On the same validation dataset, we previously found that the manual PubMed Publication Type index had recall of 0.94 and precision of 0.56 for retrieving RCTs.^4^

#### Identifying studies conducted in humans

We next remove any RCTs not conducted in humans (eg, animal or agricultural studies) using a support vector machine model, following the method described by Cohen et al.^9^ Here, we trained a similar model using abstracts of RCTs from PubMed, with labels derived as a function of whether the MeSH term “Humans” had been applied or not. We used 350 364 abstracts for training, and evaluated performance on 116 789 withheld abstracts (25% of the dataset), and evaluated using 1000 bootstrap iterations. We add all studies determined to be both RCTs and in humans to the database; these go forward for (automated) annotation.

### Automated annotation of RCTs

#### Sample size extraction

The number of participants may vary at different time points in a trial (eg, owing to withdrawals/dropouts). Here, we define sample size as the number of people randomized (and not the number of people who complete the trial). We assume that if the sample size is reported in the abstract, it will be as an integer (either in numerals or words). We first use a series of heuristics and regular expressions to convert numbers expressed in natural language to numerals (eg, “one hundred and twelve” would be replaced with “112”). We then use a multilayer perceptron model to estimate the probability that each integer represents the study sample size. This model uses a set of features describing the integer context in the abstract. These include word embeddings of adjacent tokens, initialized to weights from a word2vec model^20^ pretrained over a large corpus of PubMed articles^21^; inferred part-of-speech tags for surrounding words; and bespoke features that indicate, for example, whether some variant of the word “patients” occurs near the token under consideration.

The model was trained using 8935 RCT abstracts, for which the true number of people randomized was available: 6315 of these labels were derived automatically using registry data from ClinicalTrials.gov; the remaining 2620 were labelled manually by members of our team. We provide the complete feature set and other model details in the Supplementary Appendix.

#### PICO extraction

The penultimate step in our pipeline entails extracting snippets from abstracts that describe the PICO elements in the trial being described. We then automatically map these snippets to concept unique MeSH identifiers. Here, as in prior work, we do not distinguish between interventions and comparators (and, for example, consider “placebo” or “usual care” to be additional intervention arms)—in the rest of this article, we describe both active and control interventions as interventions.^11^ To extract the text snippets, we use a modelling approach we have described in detail previously,^12^ and summarize here. We use a long short-term memory conditional random field (LSTM-CRF)^22^ to make token-level predictions.

In this model, input texts (represented as token and character-level embeddings) are passed through an LSTM layer to yield contextualized representations of words. The token embeddings were initialized using pretrained word vectors.^21^ These representations are then passed through a CRF layer, which makes predictions as to whether each word is a population, intervention, or outcome. We trained the model on the ∼5000 RCT abstracts that comprise the EBM-NLP dataset.^12^ This dataset contains abstracts with manually annotated text snippets describing each element of the PICO.

As a second step, we expand any abbreviations within the extracted text snippets by algorithmically identifying abbreviation definitions (eg, “Atrial Fibrillation (AF)”) that appear elsewhere in the text.^23^

Finally, we follow the method set out by Demner-Fushman et al,^17^ who created the MetaMap Lite software. Briefly, a database of synonyms for medical terms is automatically compiled by retrieving all the free-text descriptions of the concepts included in vocabularies contained in the Unified Medical Language System Metathesaurus. These synonyms are minimally pre-processed (including, for example, removing database tags such as “[NOS],” and lowercasing all terms). The end result is an index of synonyms for each MeSH term. These synonyms can be sought in the extracted text snippet describing the trial population to find associated MeSH terms.

We use a different set of vocabularies compared with MetaMap Lite to generate our synonyms (namely SNOMED CT [Systematized Nomenclature of Medicine Clinical Terms], RxNorm, MeSH, MedDRA [Medical Dictionary for Regulatory Activities], and the World Health Organization ATC [Anatomic Therapeutic Chemical] classification system). These are also the vocabularies chosen by the Cochrane linked data project (https://linkeddata.cochrane.org/), specifically designed to describe PICO attributes from clinical trial reports in Cochrane Reviews, and we found that they gave good coverage for PICO concepts during development. We evaluate these PICO concept extraction steps against 1437 abstracts that had been manually labelled by the Cochrane Crowd project with structured PICO terms. Our main evaluation assesses whether the system produces exact matches to the manual terms (which we label the “strict” evaluation).

The Unified Medical Language System Metathesaurus encodes conceptual relationships between terms (eg, noting that “Anterior Wall Myocardial Infarction” is related to “Myocardial Infarction”, the latter being the parent term). Given that adjacent terms are often sufficiently close in meaning for practical purposes, we also report a “relaxed” evaluation, which in addition to exact matches considers immediate parent and child terms as correct.

#### Risk of bias

A key task in evidence-based medicine is determining whether problems in study methods might bias the results. We have previously described in detail methods for automatically assessing the risk of bias in full text RCTs (in PDF form) for the purposes of conducting a systematic review.^13–15^ In this prior work, we predicted whether studies’ results might be at risk of bias using the first version of the Cochrane Risk of Bias tool (which examines whether bias is likely due to problems in the random sequence generation, allocation concealment, and blinding, among other issues) (we note that a second version of the Risk of Bias tool has been released; we stick with the first version, owing to the availability of training data).^24^

For the Trialstreamer database, we adjust our approach in 2 ways. First, we consider abstracts only (using 57 144 abstracts of RCTs that had been manually assessed using the Cochrane Risk of Bias tool in systematic reviews in the Cochrane Library). In related work, Millard et al^16^ found that predicting risks of bias from abstracts was possible, though modestly less accurately than using full-texts. Second, here we seek to use risk of bias predictions as an input to inform search rankings, as compared with our prior work in which we aimed to produce a definitive prediction for each bias domain for use in a systematic review (after human review).

For Trialstreamer, making predictions for particular subcategories of bias, (say, asserting that the random sequence was adequately generated) is likely to be misleading to the user, given that this information is often not explicitly in the abstract. Instead, we prefer to generate a score for an overall risk of bias, using the rule of thumb outlined in the Cochrane Handbook.^25^ Using this approach, studies are rated as being at “low” risk of bias overall, when all the subcategories assessed are rated “low.” If 1 or more subcategories are rated high or unclear, the overall risk of bias is rated high or unclear.

Because our goal was to produce well-calibrated scores, we used a logistic regression model. The article title and abstract were represented in bag-of-words form with unigram, bigram, and trigram features. We used L2 regularization.^26^

We trained this model using the free text from the title and abstract of 51 429 articles and their associated manual “overall” bias assessments as labels; we evaluated the model on 5715 withheld articles. We calculated model performance both in terms of binary predictive accuracy (precision and recall) and calibration accuracy (via a calibration curve, Brier score, C-statistic). All metrics with confidence intervals were estimated using bootstrap resampling (using 1000 iterations).^19^

### Registered study retrieval

ICTRP already contains structured and semi-structured data on the registered trials. Where ICTRP fields correspond to our target extraction items, we use the contents directly instead of undertaking the associated machine learning extraction step. We use the fields describing study design, and target sample size in this way. ICTRP also contains free-text snippets describing the population, interventions, and outcomes. We automatically extract MeSH terms describing the PICO from these snippets, using the same approach described above for PubMed abstracts. Finally, owing to a lack of training data, we currently do not predict information about biases from ICTRP records.

#### The prototype web portal

**Figure 2.**
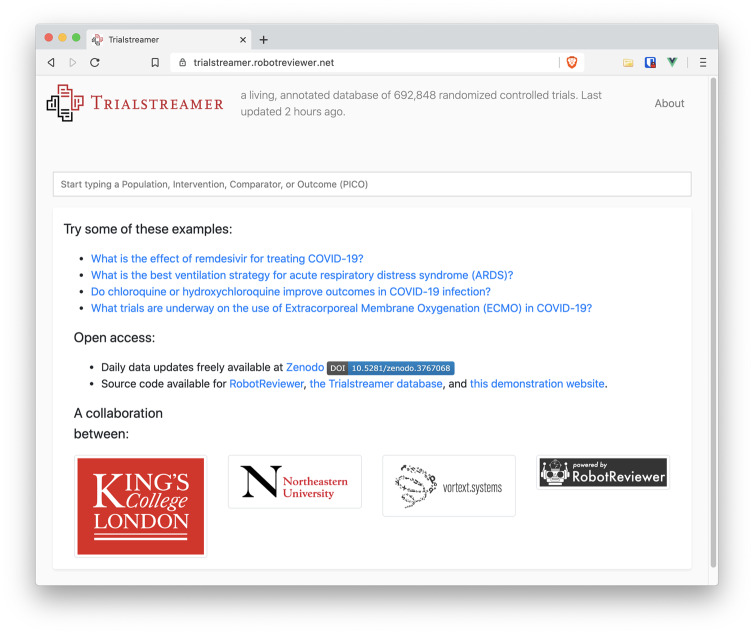
Screenshot of the Trialstreamer web interface home page.

**Figure 3.**
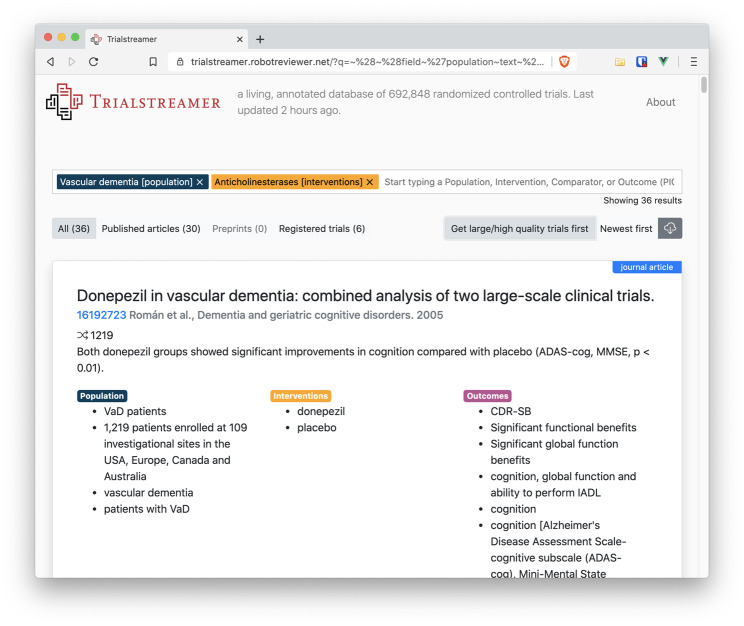
Trialstreamer search results.

We make the annotated data accessible for browsing via a web portal (https://trialstreamer.robotreviewer.net) (screenshot in Figure 2). The search portal features an autocompleter, which suggests structured terms (in population, interventions, or outcomes facets) when the user begins typing a query. For example, a physician may want to know whether anticholinesterase drugs are effective in vascular dementia. This would translate in the PICO framing to a population of people with vascular dementia, and an intervention of anticholinesterase drugs. Here, we assume that the user is interested in all potential outcomes. The user may start to type in “vascular dementia,” at which point the system suggests the corresponding concept, automatically recognizing that it is most commonly a “population.” Selecting this, the user may then begin to type a few characters of “anticholinesterases,” at which point “anticholinesterases [intervention]” is suggested. The search retrieves 30 articles (Figure 3), with the extracted information summarized for each in the search results panel.

By clicking the button labelled “Get large/high quality studies first,” search results are re-ranked, bringing to the top the largest trials that are (estimated to be) at low risk of bias. At the time of writing, the top result for this particular example is a report of 2 large RCTs (with 1219 participants) with the automatic summary showing that the trials compared donepezil vs placebo; and that donepezil led to improvement in cognition vs placebo. The Trialstreamer record includes a link to the original PubMed record. The portal also allows filtering by published trials, and registered (ie, ongoing) trials. The user may then download the search retrieval in RIS format for use in citation managers.

## RESULTS

### Validation of system performance

We summarize the performance of the system components in Table 2. The RCT classifier retrieved 94%-97% of RCT articles, with the search retrieval realizing ≥50% precision; the human study classifier had near perfect precision and recall.

**Table 2.** Summary of evaluation performance of models used in the *Trialstreamer* system

Component	Recall (95% CI)	Precision (95% CI)	C-statistic (95% CI)	Brier score (95% CI)
RCT classifier (balanced threshold)				
MeSH indexed articles	0.97 (0.96-0.98)	0.52 (0.48-0.56)	0.99 (0.98-0.99)	0.01 (0.01-0.01)
Not MeSH indexed	0.94 (0.92-0.96)	0.50 (0.44-0.57)	0.98 (0.98-0.98)	0.01 (0.01-0.01)
Human vs nonhuman classifier	1.00 (1.00-1.00)	1.00 (1.00-1.00)	0.95 (0.94-0.96)	0.003 (0.003-0.004)
Sample size extraction	0.79 (0.77-0.82)	0.88 (0.85-0.90)	n/a	n/a
PICO text spans^a^				
Population (n = 200)	0.66	0.78	n/a	n/a
Interventions (n = 200)	0.65	0.61		
Outcomes (n = 200)	0.63	0.69		
PICO concepts				
*Strict*			n/a	n/a
Population (n = 1107)	0.73 (0.70-0.75)	0.28 (0.26-0.29)		
Interventions (n = 954)	0.78 (0.75-0.80)	0.52 (0.49-0.55)		
Outcomes (n = 503)	0.64 (0.61-0.68)	0.25 (0.23-0.27)		
*Relaxed*				
Population (n = 1107)	0.78 (0.76-0.81)	0.30 (0.29-0.32)		
Interventions (n = 954)	0.85 (0.82, 0.87)	0.57 (0.54-0.60)		
Outcomes (n = 503)	0.65 (0.62-0.69)	0.30 (0.27-0.33)		
Risk of bias (probability of being at low risk of bias)	0.46 (0.40-0.52)	0.44 (0.41-0.48)	0.80 (0.79-0.81)	0.10 (0.10-0.11)

MeSH: Medical Subject Headings; n/a: Not applicable; PICO: populations, interventions/comparators, and outcomes; RCT: randomized controlled trial.

a The PICO text span accuracy results are taken from Nye et al,^12^ and we present them here for convenience.

We use the model for identifying PICO spans directly from Nye et al.^12^ This evaluation found F1 scores of 0.71, 0.65, and 0.63 for tagging individual words as describing populations, interventions, and outcomes, respectively. These are per-word (or “token”) metrics and are therefore pessimistic; extracted snippets that do not perfectly align with reference span annotations may still be high quality, and anecdotally we find that this is often the case.

We found that MeSH labels for PICO concepts had good recall for populations and interventions (recall of 0.78 and 0.85, respectively) but lower precision (0.28 and 0.52, respectively). We found that many of the apparent errors in precision could be regarded as correct, and were due to the high specificity of the Cochrane Crowd dataset used in the evaluation (in which typically only the most important concept was labeled in a study). For example, the most common false positive terms in our evaluation for population were “Patients” (8%) and “Elderly” (3%); the most common false positive intervention labels were “Placebo” (10%) and “Therapeutic procedure” (3%). We therefore conducted a post hoc manual re-evaluation of the precision for population and interventions labels, where one author not involved in the system development (R.M., a medical doctor) reassessed a random sample of 100 abstracts with their predicted labels. This evaluation found a precision of 0.79 for population descriptors and 0.84 for intervention descriptors.

**Figure 4.**
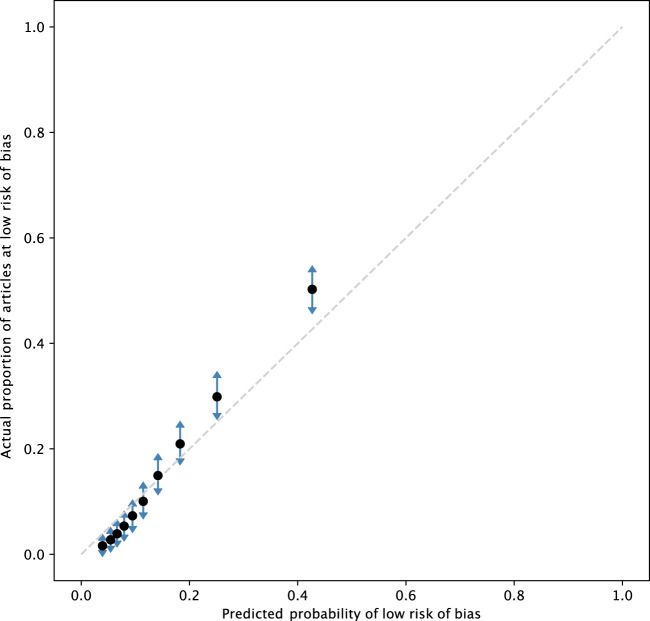
Calibration plot of the risk of bias model.

The model for predicting whether an RCT was at low risk of bias showed good calibration and ranking ability (Brier score = 0.10, C-statistic = 0.80) (see calibration plot in Figure 4). Binary classification was less accurate with regard to the gold standard labels (F1 = 0.45).

### Database contents

**Figure 5.**
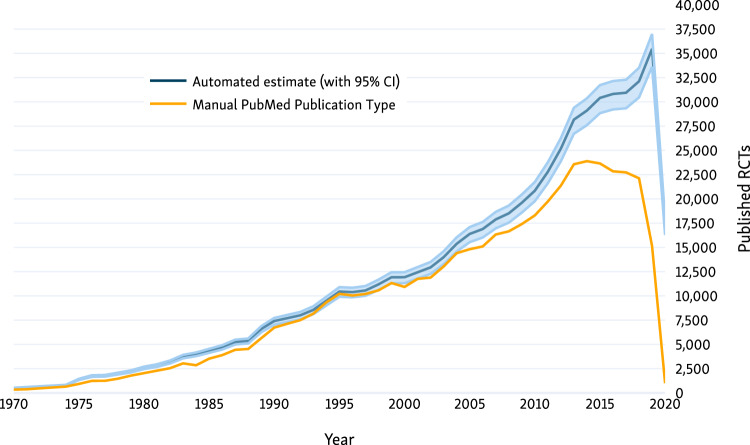
Counts of all randomized controlled trials (RCTs) in PubMed, estimated by manual indexing (yellow) vs automation (blue). CI: confidence interval.

**Figure 6.**
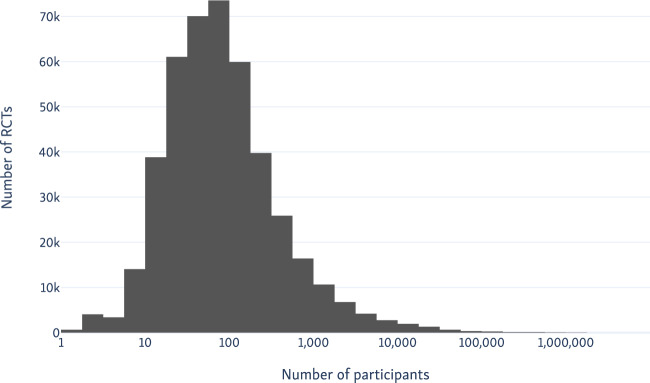
Histogram of the number of trial participants in all randomized controlled trials (RCTs) in PubMed, as extracted by our sample size extraction model.

On June 6, 2020, there were 673 191 RCTs indexed in the Trialstreamer database; 22 363 of these RCTs had been published in the first 5 months of 2020 (compared with 1319 manually indexed in PubMed). This equates to 142 RCTs published daily in the same period. We compare the manual indexing in PubMed vs the automated system in Figure 5. This figure shows that the numbers of trials we identify are similar to those done manually by PubMed until approximately 2013, but thereafter, the number of trials identified manually by PubMed plateaus, then falls sharply from 2018 onward. In total, the system identified 178 830 additional records that had not been tagged as RCTs in PubMed. A total of 49% of these additional records were published in the past 5 years. We present the distribution of sample sizes in the trials in Figure 6. The median sample size was 66 (interquartile range, 30-181). We additionally include 303 319 records from ICTRP.

## DISCUSSION

At present, accessing all RCTs in humans relevant to a particular query or clinical question is difficult for a few key reasons. First, if one relies on manual indexing of RCTs (eg, relying on the Publication Type tag in PubMed), this will necessarily constitute an incomplete subset due to the lag inherent to manual categorization (Figure 5), particularly leading to the most recent research being missed. Second, trials are most commonly reported in unstructured free text, which means that retrieving those relevant to a specific question (or PICO frame) is nontrivial. Further, even once relevant trials are retrieved, one must manually appraise the quality of trials, extract sample sizes, and peruse the text to infer what the reported results are. All of this takes time, a problem exacerbated by the rapid expansion of the biomedical literature.

Trialstreamer uses machine learning and natural language processing to address these issues, in turn providing a new publicly available resource that we make available to the broader community: a continuously updated, comprehensive database of all published RCTs in humans, with semi-structured data extracted for all of them. This resource may facilitate additional research (eg, allowing one to investigate the clinical topics for which there exists a paucity of RCT evidence), evidence scoping (or “rapid reviews”), and clinical question answering for healthcare practitioners interested in efficiently seeking out new trial results related to a specific question.

No machine learning models have perfect accuracy, and we have made some pragmatic decisions around the use of models, which have strengths and limitations. Our system recalls 97% of RCTs (as compared with the standard used in systematic review search filters, which achieve ≈98.5% recall).^4^ However, we trade off this small fraction of missed articles for substantially higher precision, which should allow the data to be used for real-time question answering or scoping, without the need for extensive manual filtering. Likewise, the system currently monitors only PubMed for publications; although PubMed does include the vast majority of relevant clinical trials, a small proportion would only be found on searching additional databases.

Likewise, our model for assessing the risk of bias has, on face value, poor performance for binary classification (given recall of 0.45, a substantial portion of high quality studies would be missed in a binary classification mode). However, the model probabilities show good calibration, and thus are useful in the current system as a means of ranking documents, and surfacing the highest-quality evidence first.

In future work, we plan improvements to the system, particularly on expanding sources of data (to include preprint servers and other sources of trial data) improving the accuracy of the data extraction models, and adding structured information on the trial results (both automatically extracted from the text and linked from structured sources such as ClinicalTrials.gov). We additionally plan to evaluate how well the system works for rapid answering of a variety of real clinical questions and evidence synthesis tasks.

## CONCLUSION

We have introduced Trialstreamer, a living, continuously updated database of semi-structured information automatically extracted from all published clinical trials as they are published. We provided quantitative evaluations of all individual components comprising Trialstreamer. We make both the underlying database available, as well as a public web interface that facilitates search. Additionally, all code (including model implementations, database creation scripts, and our prototype) for Trialstreamer is open source. We hope that the database will be a valuable resource for the informatics community, in addition to clinicians and health researchers.

## FUNDING

IJM is supported by the UK Medical Research Council, through its Skills Development Fellowship program, fellowship MR/N015185/1. This work is funded by the National Institutes of Health under the National Library of Medicine, grant R01-LM012086, “Semi-Automating Data Extraction for Systematic Reviews.”

## AUTHOR CONTRIBUTIONS

IJM and BCW conceived the study, with input from JT, RMar, AN, and FS. AN-S, JT, BN, IJM, AN, and BCW were involved in creation of the training and evaluation data. IJM, BN, and BCW were involved in coding and accuracy evaluation of machine learning models. RMac was involved in manual model evaluation. IJM, JK, and BCW were involved in coding of the web portal. IJM and BCW wrote the initial draft of the manuscript. All authors were involved in making critical revisions of manuscript and approving the final version.

## SUPPLEMENTARY MATERIAL

Supplementary material is available at Journal of the American Medical Informatics Association online.

## Supplementary Material

ocaa163_supplementary_data

## References

[1] Chalmers I. The Cochrane Collaboration: preparing, maintaining, and disseminating systematic reviews of the effects of health care. Ann N Y Acad Sci 1993; 703: 156–65. 8192293 10.1111/j.1749-6632.1993.tb26345.x

[2] Shaughnessy AF, Slawson DC, Bennett JH. Becoming an information master: a guidebook to the medical information jungle. J Fam Pract 1994; 39: 489–99. 7964548

[3] Bastian H, Glasziou P, Chalmers I. Seventy-five trials and eleven systematic reviews a day: how will we ever keep up? PLoS Med 2010; 7 (9): e1000326. 20877712 10.1371/journal.pmed.1000326 PMC2943439

[4] Marshall IJ, Noel-Storr A, Kuiper J, et al Machine learning for identifying randomized controlled trials: an evaluation and practitioner’s guide. Res Synth Methods 2018; 9 (4): 602–14. doi: 10.1002/jrsm.1287. 10.1002/jrsm.1287 PMC6030513 29314757

[5] Thomas J, Kneale D, McKenzie JE, et al Chapter 2: determining the scope of the review and the questions it will address In: Higgins JPT, Thomas J, Chandler J, et al, eds. Cochrane Handbook for Systematic Reviews of Interventions. Version 6.0. London: Cochrane Collaboration; 2019.

[6] Marshall IJ, Wallace BC. Toward systematic review automation: a practical guide to using machine learning tools in research synthesis. Syst Rev 2019; 8 (1): 163. 31296265 10.1186/s13643-019-1074-9 PMC6621996

[7] Cohen AM, Smalheiser NR, McDonagh MS, et al Automated confidence ranked classification of randomized controlled trial articles: an aid to evidence-based medicine. J Am Med Inform Assoc 2015; 22 (3): 707–17. 25656516 10.1093/jamia/ocu025 PMC4457112

[8] Wallace BC, Noel-Storr A, Marshall IJ, et al Identifying reports of randomized controlled trials (RCTs) via a hybrid machine learning and crowdsourcing approach. J Am Med Inform Assoc 2017; 24 (6): 1165–8. 28541493 10.1093/jamia/ocx053 PMC5975623

[9] Cohen AM, Dunivin ZO, Smalheiser NR. A probabilistic automated tagger to identify human-related publications. Database 2018; 2018. doi: 10.1093/database/bay079. 10.1093/database/bay079 PMC6146117 30184195

[10] Summerscales RL, Argamon S, Bai S, et al Automatic summarization of results from clinical trials. Proceedings (IEEE Int Conf Bioinformatics Biomed) 2011; 2011: 372–7. doi: 10.1109/BIBM.2011.72.

[11] Wallace BC, Kuiper J, Sharma A, et al Extracting PICO sentences from clinical trial reports using supervised. J Mach Learn Res 2016; 17: 1–25. PMC5065023 27746703

[12] Nye B, Jessy Li J, Patel R, et al A corpus with multi-level annotations of patients, interventions and outcomes to support language processing for medical literature. Proc Conf Assoc Comput Linguist Meet 2018; 2018: 197–207. 30305770 PMC6174533

[13] Marshall IJ, Kuiper J, Wallace BC. Automating risk of bias assessment for clinical trials. In: proceedings of the 5th ACM Conference on Bioinformatics, Computational Biology, and Health Informatics; 2014: 88–95. 10.1109/JBHI.2015.2431314 25966488

[14] Marshall IJ, Kuiper J, Wallace BC. RobotReviewer: evaluation of a system for automatically assessing bias in clinical trials. J Am Med Inform Assoc 2016; 23 (1): 193–201. 26104742 10.1093/jamia/ocv044 PMC4713900

[15] Zhang Y, Marshall I, Wallace BC. Rationale-augmented convolutional neural networks for text classification. *Proc Conf Empr Methods Nat Lang Process* 2016; 2016: 795–804. https://www.ncbi.nlm.nih.gov/pubmed/28191551. 10.18653/v1/d16-1076 PMC5300751 28191551

[16] Millard LA, Flach PA, Higgins JP. Machine learning to assist risk-of-bias assessments in systematic reviews. Int J Epidemiol 2016; 45 (1): 266–77. 26659355 10.1093/ije/dyv306 PMC4795562

[17] Demner-Fushman D, Rogers WJ, Aronson AR. MetaMap Lite: an evaluation of a new Java implementation of MetaMap. J Am Med Inform Assoc 2017; 24 (4): 841–4. 28130331 10.1093/jamia/ocw177 PMC6080672

[18] Montori VM, Wilczynski NL, Morgan D, et al Optimal search strategies for retrieving systematic reviews from Medline: analytical survey. BMJ 2005; 330 (7482): 68. 15619601 10.1136/bmj.38336.804167.47 PMC543864

[19] Steyerberg EW, Harrell FE Jr, Borsboom GJ, et al Internal validation of predictive models: efficiency of some procedures. J Clin Epidemiol 2001; 54 (8): 774–81. 11470385 10.1016/s0895-4356(01)00341-9

[20] Mikolov T, Sutskever I, Chen K, et al Distributed representations of words and phrases and their compositionality In: Burges CJC, Bottou L, Welling M, et al, eds. Advances in Neural Information Processing Systems 26. Red Hook, NY: Curran Associates; 2013: 3111–9.

[21] Pyysalo S, Ginter F, Moen H, et al Distributional semantics resources for biomedical text processing. In: proceedings of the LBM; 2013.

[22] Huang Z, Xu W, Yu K. Bidirectional LSTM-CRF models for sequence tagging. arXiv:1508.01991 Cs; 2015.

[23] Schwartz AS, Hearst MA. A simple algorithm for identifying abbreviation definitions in biomedical text. Pac Symp Biocomput 2003; 2003: 451–62. 12603049

[24] Higgins JPT, Altman DG, Gotzsche PC, Cochrane Bias Methods Group, et al The Cochrane Collaboration’s tool for assessing risk of bias in randomised trials. BMJ 2011; 343: d5928. 22008217 10.1136/bmj.d5928 PMC3196245

[25] Higgins JPT, Deeks JJ. Selecting studies and collecting data In: Higgins JPT, Green S, eds. Cochrane Handbook for Systematic Reviews of Interventions. Version 5.1.0. New York: Wiley; 2008: 7.1–7.28.

[26] Ng AY. Feature selection, L 1 vs. L 2 regularization, and rotational invariance. In: proceedings of the Twenty-First International Conference on Machine Learning (ICML ’04); 2004.

