# Supplementary material for: Trialstreamer: A living, automatically updated database of clinical trial reports
Source: J Am Med Inform Assoc. 2020 Sep 17;27(12):1903–12. doi: 10.1093/jamia/ocaa163 (PMC7727361; doi:10.1093/jamia/ocaa163)
Supplement: ocaa163_supplementary_data [file ocaa163_supplementary_data.docx]

# Appendix

**Sample size extraction model details**

We provide additional details regarding our sample size extraction model, as this model has not been described in previous work.

To classify a given word (integer) at index *t* in the abstract, the model consumes as inputs the two words to its left and the two to its right (i.e., the words at position *t*-2 and *t*-1, and those at *t*+1 and *t*+2). Words are passed through an embedding layer and mapped to distributed representations (vectors). We initialize this to 200-dimensional word vectors pre-trained via *word2vec*[*^7^*](https://paperpile.com/c/RBlYyb/6AUc) over a large corpus of PubMed articles.[^8^](https://paperpile.com/c/RBlYyb/gKHB) Embeddings are fine-tuned (i.e., updated via backpropagation) during training. We concatenate the embeddings corresponding to these four surrounding words.

As additional features, we use predicted part of speech (P.O.S.) tags for the words immediately surrounding the target word; we make these predictions via the Spacy library (v 2.0.12). P.O.S. tags are encoded as one-hot vectors indicating which of the 56 P.O.S. types defined in Spacy were predicted for the tokens immediately adjacent to the target word.

In addition to the above we use a few bespoke features derived for the sample size task. These include whether the word “year” or “years” occurs within five tokens of the target word (years often “look like” sample sizes), and whether “patients” or a variant thereof (“subjects”, “participants”; case invariant) occurs within four tokens of the target word (a strong indicator that a target is a sample size). We also include a feature that indicates whether the token under consideration is the largest integer in the abstract, and whether it is an integer between 1940 and 2020 (a somewhat arbitrary range that may suggest the integer refers to a year). All feature engineering was performed independently of the test set, i.e., before we evaluated the model.

The model architecture itself is a simple feed-forward neural network (Multi-Layer Perceptron). The above inputs are concatenated (yielding 916 dimensions) and passed forward through a fully-connected dense layer with 128-dimensional outputs, followed by an element-wise ReLU activation function.[^9^](https://paperpile.com/c/RBlYyb/NA0k) These outputs are passed through a second fully connected layer that yields a 64-dimensional output, again followed by a ReLU. Finally, this is passed to a single dimensional layer with a sigmoid activation, yielding a predicted probability that the token under consideration describes a sample size. During training we impose dropout[^10^](https://paperpile.com/c/RBlYyb/nXvs) between the hidden dense layers (with dropout probability equal to 0.2) as a regularization strategy. We used the Adam optimizer with the default parameters in the Keras (v 2.1.5) implementation.
